# Unravelling the myth: A cross-sectional study on coffee consumption among anesthesiologists in France

**DOI:** 10.1097/MD.0000000000040762

**Published:** 2024-11-29

**Authors:** Mohammad Barnawi, Duaa Alawadi, Khalid Alzahrani, Ramy Samargandi, Mariam Boutros

**Affiliations:** a Department of Surgery, Faculty of Medicine, Al-Baha University, Alaqiq, Saudi Arabia; b Department of Anesthesia and Surgical ICU, Caen University Hospital, Caen, France; c Department of Anesthesia, King Salman Bin Abdulaziz Medical City, Al-Madinah Al-Munawwarah, Saudi Arabia; d Department of Surgery, Faculty of Medicine, Jeddah University, Jeddah, Saudi Arabia.

**Keywords:** anesthesia, caffeine consumption, Certified Registered Nurse Anesthetists, coffee, France

## Abstract

It is a common preconception in France that anesthesiologists consume the largest amounts of coffee. This study is aimed to evaluate the relation between working in anesthesiology and increased coffee consumption. Cross-sectional, multicentric study carried out on anesthesiology professionals in France including anesthesiologists (residents and seniors) and Certified Registered Nurse Anesthetists (nurses and students). We performed a paper-based and a web-based survey among participants in the period from January 31, 2020, until March 31, 2020. Data were recorded for each participant including, demographics, function, place of work, and details of coffee consumption. Coffee consumption. We considered the hypothesis to be statistically significant if *P* < .01. *F* test was performed to evaluate the variance between different populations. A total of 690 anesthesiology professionals participated in this survey. Average caffeine consumption within anesthesiology team is 308 ± 168 mg/d in the daily operating room and 359 ± 200 in on-call duties. The is a statistically significant increase in caffeine consumption during work in anesthesiology (*P* < .001). Evolution of coffee consumption were more important in daily operating room than in on-call duties with *F* test of 118.54 and 205.14, respectively. The majority of respondents (74%) enjoyed coffee for pleasure, while 12% drank it to boost alertness. However, 71% noticed no improvement in attention after consumption. Caffeine dependence symptoms were seen in only 5% of all respondents and 11% of those who consumed 600 mg or more of caffeine daily. Working in the field of anesthesiology seems to be associated with increased coffee consumption, however average coffee consumption among anesthesiologist in France is considered safe. These findings underscore the importance of carefully managing working conditions, shift schedules, and working hours for anesthesiologists to lessen potential risks associated with excessive caffeine intake.

## 1. Introduction

Caffeine, chemically identified as 1,3,7-trimethylxanthine, serves as the primary active component in coffee. Its absorption primarily takes place in the small intestine, accounting for approximately 80%, with a lesser degree of absorption occurring in the stomach, around 20%. This absorption process is analogous in most studied species.^[1,2]^ Coffee is one of the most widespread beverages in human history, and its consumption constitutes an important part of the daily lives of people all over the world as a major source of caffeine for most populations.^[3,4]^ Scandinavian countries are considered as the Europe’s highest coffee consumers in 2018. Finland is in the first position with 12 kg/person/year, and France is eighteenth in coffee consumption with about 5.4 kg/person/year. The average consumption of the French population is around one and half cup of coffee per day (0.6 cups/d).^[5,6]^ One cup of coffee is equivalent to approximately 90 mg of caffeine. Most of the literature considers 1 cup of coffee contains roughly 90 mg of caffeine. According to the majority of existing literature,^[6–8]^ caffeine consumption is categorized as follows: Low caffeine consumption: Up to 200 mg/d or 1–2 cups/d. Moderate caffeine consumption: around 400 mg/d or 3 to 5 cups/d. High coffee consumption: At or above 600 mg/d or 6–10 cups/d. A systematic review by Doepker et al,^[7]^ published in 2018 concluded that caffeine is not associated with adverse effects: 400 mg/d for healthy adults, 300 mg/d for pregnant women, and 2.5 mg/kg body weight/day for adolescents and children. These levels remain appropriate for 5 outcome areas (acute toxicity, cardiovascular toxicity, bone and calcium effects, behavior, and development and reproduction).^[7]^ The health benefits and risks of caffeine consumption have long been a subject of debate. Some studies suggest potential reductions in the risk of various diseases^[8,9]^ and mortality,^[10]^ while others have indicated a potential increase in cardiovascular risk.^[11]^ Excessive caffeine intake can result in tachycardia, hypertension, and a heightened risk of cardiac arrhythmias. These effects can be particularly concerning for individuals with underlying heart conditions or those predisposed to cardiovascular issues.^[12]^ Another potential risk associated with heavy caffeine consumption is its impact on the central nervous system. excessive consumption can lead to overstimulation, resulting in symptoms such as anxiety, restlessness, nervousness, and, in some cases, panic attacks. Furthermore, it can disrupt sleep patterns and contribute to insomnia, affecting overall mental well-being and cognitive function.^[13]^

Working in the healthcare system often entails extended periods of wakefulness and dealing with fatigue, especially during night shifts. Anesthesiologists are required to be alert, knowledgeable, and proficient in responding to clinical challenges while maintaining prolonged vigilance to ensure the safe administration of anesthesia and critical care. Common strategies employed to stay vigilant and awake at work include the consumption of coffee and taking short naps.

Studies have demonstrated that caffeine, typically consumed at doses between 200 and 600 mg, can temporarily enhance vigilance and performance.^[14]^ In fact, a driving study found that after consuming coffee, 75% of individuals performed as well during nocturnal driving as they did during daytime driving, whereas after a nap, this figure was 66%. In contrast, after a placebo (decaffeinated coffee with no nap), only 13% performed at an equivalent level.^[15]^ According to Bonnet et al,^[16]^ caffeine is generally the most effective countermeasure for improving night-shift alertness for health professionals.

In France, there is a prevalent belief that anesthesiologists consume significant amounts of coffee to help them cope with the demands of their work. Therefore, the aim of this study is to examine the patterns of caffeine consumption among professionals working in the field of anesthesiology in France.

## 2. Materials and methods

A cross-sectional, multicentric, observational study, conducted from January 31, 2020, to March 31, 2020. It was carried out within the anesthesiology community, including anesthesiologists (both residents and senior practitioners) and Certified Registered Nurse Anesthetists (comprising both students and registered nurse anesthetists). The primary objective of our study is to evaluate the relationship between caffeine consumption and working in anesthesiology, with a secondary focus on variations in caffeine intake based on different working conditions. Additionally, we conducted an analysis to compare caffeine consumption before and after working in anesthesiology. We conducted surveys using 2 methods: in-person interviews or online questionnaires distributed via respondents’ professional email addresses. Throughout the data collection process, we implemented rigorous measures to ensure respondent anonymity, thereby safeguarding their privacy and maintaining the confidentiality of their responses.

Exclusion criteria included individuals who declined to participate and those from specialties other than anesthesia. We collected comprehensive data for each participant, containing demographics, professional roles, workplace locations, and details about their coffee consumption patterns before and after joining the field of anesthesiology.

The ethical approval has been waived by the Clinical Research Department of Caen University Hospital due to the cross-sectional nature of the study and the use of anonymized data. The consent was obtained from each participant at the beginning of the questionnaire. Continuous data were presented as mean ± standard deviation, while categorical data were expressed as percentages. Statistical analyses were conducted using SPSS® software (Version 23.0, Armonk, NY: IBM Corp). Univariate analysis of quantitative data was carried out using the *t* test (ANOVA). We considered the hypothesis statistically significant if *P* < .01. The *F* test was utilized to assess variance differences among various populations.

## 3. Results

A total of 690 professionals participated in the survey, with 61% (n = 423) of the respondents being females and 77.2% (n = 533) being Certified Registered Nurse Anesthetists. Most respondents, accounting for 53.6% (n = 370), work in university hospitals. A significant portion, 44.3% (n = 306) of the participants, belonged to the age group ranging from 30 to 39 years old. The detail of the respondents is outlined in Table 1.

**Table 1:** Demographic data of study participants.

Sociodemographic data		*n*	%
Gender	Male	267	39%
	Female	423	61%
Age grouping	<30	96	14%
	30–39	306	44%
	40–49	200	29%
	50–59	73	11%
	>60	15	2%
Place of work	University Hospital	370	54%
	Peripheral Hospital	245	35%
	Private Hospital	75	11%
Position	Senior	37	5.4%
	Junior	49	7.1%
	CRNA	533	77.2%
	Student CRNA	71	10.3%
Reason of drinking	Pleasure	514	74.5%
	Vigilance	87	12.6%
	Dependence	40	5.8%
	Other	49	7.1%

CRNA = Certified Registered Nurse Anesthetists.

In our study, we found that the average caffeine consumption within the anesthesiology team was moderate, with levels of 308 ± 168 mg/d in the daytime operating room and 359 ± 200 mg/d during on-call duties. The majority of anesthesiology professionals fell into the category of moderate coffee consumers, comprising 49% (n = 339) in the daytime operating room and 46% (n = 318) during on-call duties. Only 8.8% (n = 61) of the anesthesiologists were considered high coffee consumers in the daytime operating room, and 22% (n = 152) during on-call duties, with a daily coffee consumption exceeding 6 cups. Table 2 provides an overview of general coffee consumption by cups among respondents before and after joining the field of anesthesiology.

**Table 2:** Distribution of coffee drinkers before and after working in anesthesiology.

	0 cup/d	1–2 cup/d	3–5 cup/d	>6 cup/d
Before	146	266	232	46
OR	93	197	339	61
On-call	98	122	318	152
Day off	145	345	180	20

OR = operating room.

**Figure 1.**
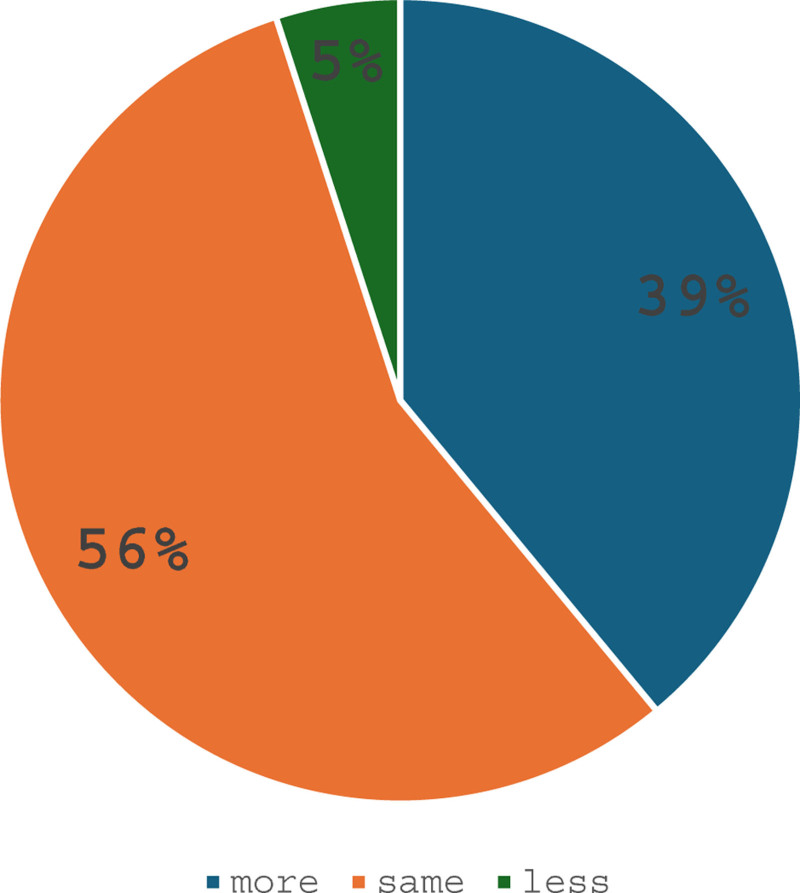
What respondents think about personal coffee evolution.

Among the respondents, 21% (n = 146) had not been coffee drinkers before joining the field of anesthesiology, while 13% (n = 93) had never been coffee drinkers at all. There was a statistical significance relation between increased caffeine consumption and working in anesthesiology (*P* < .001). This relation was more pronounced in the daily operating room setting compared to on-call duties, with an *F* test value of 118.54 and 205.14, respectively, Table 3 presents descriptive data regarding caffeine consumption in our study population. 56% of respondents believed that their coffee consumption remained unchanged after joining the field of anesthesiology. Figure 1 summarizes the participants’ perceptions regarding changes in their personal coffee consumption over time. According to 44% of the respondents, the leading cause of increased coffee consumption was sleep deprivation, followed by a demanding daily operating room schedule and the influence of colleagues who were frequent coffee drinkers. Out of the respondents, 74% (n = 514) enjoyed coffee for its pleasurable aspects, while 12% (n = 87) sought it to enhance vigilance. Interestingly, despite this, 71% (n = 490) of the respondents reported no noticeable increase in attention or vigilance after coffee consumption. Signs of caffeine dependence, such as headaches, nervousness, and fatigue, were observed in only 5% of all respondents and 11% of high caffeine consumers who consumed roughly 600 mg of caffeine per day or more.

**Table 3:** Descriptive data of caffeine consumption.

Category	Groups	*n*	Mean (mg/d)	SD	95% CI	ANOVA	
						*F*	*P*
OR	0†	470	213.62	231.399	192.64–234.59	118.549	<.001
	1*	220	393.64	118.420	377.90–409.37		
	Total	690	271.01	218.984	254.65–287.38		
On-call	0†	366	224.59	251.999	433.31–461.75	205.149	<.001
	1*	324	447.53	130.111	434.10–462.18		
	Total	690	329.28	232.325	311.91–346.64		

OR = operating room.

* Increase in caffeine consumption.

† No evolution in caffeine consumption or decrease in caffeine consumption.

## 4. Discussion

Working in anesthesiology requires a constant state of vigilance, which often leads to an increase in daily caffeine consumption. Our study revealed that the average daily coffee consumption among anesthesiology professionals working in France is approximately 4 to 5 cups/d, equivalent to approximately 400 mg of caffeine daily. This level of consumption is nearly 3 times higher than the average caffeine intake in the general French population, which is around 155 mg of caffeine per day.^[6]^The results of our study are consistent with the findings regarding coffee consumption among Scandinavian anesthesiologists as reported by Do et al,^[17]^ where the average intake was 4 cups/d. This level of consumption stands in stark contrast to the Swiss study on anesthesiologists’ coffee purchasing patterns at work, which reported a mere 39 cups annually.^[18]^ This discrepancy may be due to differences in coffee drinking habits between anesthesiologist in France and Switzerland, or to the fact that the Swiss study only measured coffee purchased at work, while the other studies measured total coffee consumption regardless of location.

It is important to note that such a coffee consumption level, around 4 to 5 cups/d or approximately 400 mg of caffeine daily, is generally considered safe and is often associated with more benefits than harm. Research indicates that it may even lead to a 10% lower risk of total mortality.^[19]^ In our study, we found that high coffee consumption was significantly more common during on-call duties (22%) compared to daytime operating room shifts (8.8%). This suggests that the increased need for vigilance and the heavier workload during emergency cases may contribute to higher caffeine intake. This specific correlation between coffee consumption and on-call duties has not been thoroughly explored in the existing literature. However, even though a smaller percentage of anesthesiologists consider themselves heavy coffee drinkers, it is important to recognize that research indicates a daily caffeine intake exceeding 500 mg can lead to various adverse effects. These may include increased tension, nervousness, anxiety, excitement, irritability, nausea, paranesthesia, tremors, perspiration, palpitations, restlessness, and possibly dizziness.^[20]^ Additionally, this heightened caffeine consumption can potentially impair judgment, delay and compromise responses to clinical changes, lead to poor communication, and result in suboptimal record-keeping. Therefore, it is crucial to raise awareness within this population about the potential side effects of excessive caffeine consumption.

Our study revealed that the majority of respondents drink coffee primarily for pleasure (74%), while only 6% consume it due to dependence. This suggests that coffee drinking is a widespread habit among French people, driven mainly by enjoyment rather than necessity. However, we also found that a significant number of respondents reported that the primary reason for increasing their caffeine intake was a lack of sleep the night before a workday. This indicates that while coffee is generally consumed for pleasure, it also serves as a compensatory tool for managing fatigue related to insufficient sleep. Interestingly, only 5% of participants reported experiencing caffeine-related symptoms such as headaches, nervousness, and fatigue. Nevertheless, it is important to note that we cannot definitively attribute these symptoms to caffeine consumption, as other factors—particularly in the demanding field of anesthesiology—could also contribute to these effects. The high-stress environment, irregular hours, and the intensity of the work may all play roles in these symptoms, making it difficult to isolate caffeine as the sole cause.

High caffeine consumption among anesthesiologists, particularly among those identified as heavy drinkers—who notably increase their intake during on-call duties—necessitates specific modifications to the anesthesia environment. These modifications should include optimizing shift schedules to better manage fatigue and reduce the reliance on excessive caffeine. Regular monitoring of caffeine intake is essential, supported by educational programs that raise awareness about the risks associated with heavy consumption and tools for self-assessment and health screenings to manage intake and its effects effectively.

Despite its contributions to understanding the current caffeine consumption patterns among anesthesiology professionals in France, the study has several limitations. First, the cross-sectional design does not allow for the establishment of causal relationships, as it only provides a snapshot of data at a single point in time without assessing how changes in 1 variable might influence another over time. Second, the reliance on self-reported data introduces the potential for recall bias, as participants may not accurately remember or report their caffeine consumption and related experiences.

## 5. Conclusions

In summary, this study highlights that anesthesiologists in France consume significantly more caffeine than the general population, particularly during on-call duties. While moderate caffeine intake is generally safe, high consumption can pose risks to health and job performance. Future research should focus on confirming the prevalence of high caffeine consumption, its impact on job performance, and exploring alternative alertness strategies.

## Author contributions

**Conceptualization:** Mohammad Barnawi, Duaa Alawadi, Mariam Boutros.

**Data curation:** Mohammad Barnawi, Khalid Alzahrani.

**Formal analysis:** Ramy Samargandi, Mariam Boutros.

**Supervision:** Mariam Boutros.

**Writing – review & editing:** Mohammad Barnawi, Khalid Alzahrani.

**Writing – original draft:** Duaa Alawadi, Ramy Samargandi.
